# High-hole mobility polycrystalline Ge on an insulator formed by controlling precursor atomic density for solid-phase crystallization

**DOI:** 10.1038/s41598-017-17273-6

**Published:** 2017-12-05

**Authors:** Kaoru Toko, Ryota Yoshimine, Kenta Moto, Takashi Suemasu

**Affiliations:** Institute of Applied Physics, University of Tsukuba, 1-1-1 Tennodai, Tsukuba, Ibaraki, 305-8573 Japan

## Abstract

High-carrier mobility semiconductors on insulators are essential for fabricating advanced thin-film transistors, allowing for three-dimensional integrated circuits or high-performance mobile terminals. We investigate the low-temperature (375–450 °C) solid-phase crystallization (SPC) of Ge on a glass substrate, focusing on the precursor conditions. The substrate temperature during the precursor deposition, *T*_d_, ranged from 50 to 200 °C. According to the atomic density of the precursor and the *T*_d_ dependent SPC properties, the precursor conditions were determined by three regimes: the low-density regime (*T*_d_ < 100 °C), high-density regime (100 ≤ *T*_d_ ≤ 125 °C), and nucleation regime (*T*_d_ > 125 °C). The use of the precursor in the narrow high-density regime enabled us to form SPC-Ge with a hole mobility of 340 cm^2^/Vs, the highest value among semiconductor thin films grown on insulators at low temperature (<900 °C). The origins of the high hole mobility were determined to be both a large grain size (5 µm) and a low energy barrier height (6.4 meV) for the grain boundary. The findings from and knowledge gained in this study, that is, the influence of the precursor conditions on subsequent crystal growth, will be universal and applicable to various materials.

## Introduction

Germanium has been proposed as a major candidate for next-generation electronic devices because of its high carrier mobility and good compatibility with Si^1–3^. In particular, the hole mobility of Ge is the highest among semiconductor materials, which can be used to realize high-performance complementary metal-oxide-semiconductor (CMOS) devices^3^. The developments of gate stacks and source/drain junction technologies have led to high effective mobilities in Ge metal-oxide-semiconductor field-effect-transistors (MOSFETs) exceeding those in Si-MOSFETs^4–7^. For expanding the application of Ge-MOSFETs in system-in-displays (system-on-panels) or three-dimensional integrated circuits, Ge on insulator (GOI) technology has been developed using mechanical transfer^8^, oxidation-induced condensation^9,10^, epitaxial growth on Si on insulator (SOI)^11,12^, and rapid-melting growth^13–16^. To avoid thermal damage to the substrates and to lower the process costs, the low-temperature formation (<600 °C) of GOI is necessary. Polycrystalline Ge (poly-Ge) thin films have been directly formed on glass or plastic substrates at low temperatures using solid-phase crystallization (SPC)^17–21^, laser annealing^22–24^, chemical vapor deposition (CVD)^25,26^, flash lamp annealing (FLA)^27^, and metal-induced crystallization (MIC)^28–32^. The use of these techniques has allowed researchers to fabricate Ge thin-film transistors (TFTs) via all-low-temperature processes^20,21,27,32^. The performance of the Ge-TFTs, however, has been no match for that of Si-MOSFETs. To further improve Ge-TFTs, one needs to study not only device technology but also crystallization techniques.

In 2009, we demonstrated a hole mobility of 140 cm^2^/Vs for poly-Ge on glass formed by two-step SPC of amorphous (a−) Ge at 425 °C followed by 500 °C^17^. In the last couple of years, the highest hole mobility has been updated frequently: FLA has achieved 200 cm^2^/Vs^27^, followed by Au-induced crystallization achieving 160–210 cm^2^/Vs^31,32^. On the other hand, incorporating Sn in Ge has been found to be effective for enhancing the hole mobility^33–35^. The SPC of a-GeSn has achieved a hole mobility of 130 cm^2^/Vs^34^, which was then enhanced to 320 cm^2^/Vs by controlling the thickness of the a-GeSn layer^35^. The present study demonstrates a simple method to achieve pure poly-Ge with a hole mobility of 340 cm^2^/Vs by controlling the atomic density of an a-Ge precursor. This hole mobility is the highest ever recorded for a semiconductor thin film including Ge, Si, compound, oxide, and organic materials formed on insulators at temperatures below 900 °C.

## Results

**Figure 1:**
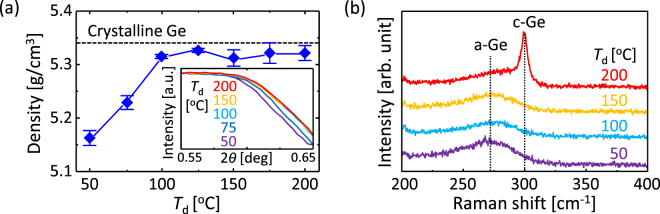
Characteristics of the as-deposited Ge layers. (**a**) Density of Ge as a function of *T*_d_. The data for crystalline Ge is shown by the dotted line. The inset shows the raw XRR patterns, focusing on the area reflecting the density. (**b**) Raman spectra for samples with *T*_d_ = 50, 100, 150, and 200 °C.

The as-deposited Ge layers, precursors for SPC, were evaluated by X-ray reflectivity (XRR) and Raman spectroscopy. Figure 1a shows that the density of the Ge layer approaches that of crystalline Ge with increasing the deposition temperature *T*_d_ and becomes nearly saturated at *T*_d_ > 100 °C. The density of the Ge layer for *T*_d_ = 50 °C almost agrees with the data from previous study on the a-Ge layer deposited without heating the substrate^36^. Figure 1b shows that samples with *T*_d_ = 50, 100, and 150 °C exhibit broad peaks near 270 cm^−1^, corresponding to a-Ge^17,18^. The sample with *T*_d_ = 200 °C exhibits a sharp peak near 300 cm^−1^, corresponding to crystalline Ge-Ge bonding^17,18^, in addition to a broad peak near 270 cm^−1^. This result indicates that the sample with *T*_d_ = 200 °C includes both crystalline and amorphous states. The optical studies suggest the following: (i) higher *T*_d_ provides denser a-Ge; and (ii) crystalline Ge nuclei start to form in the a-Ge layer at *T*_d_ > 150 °C.

**Figure 2:**
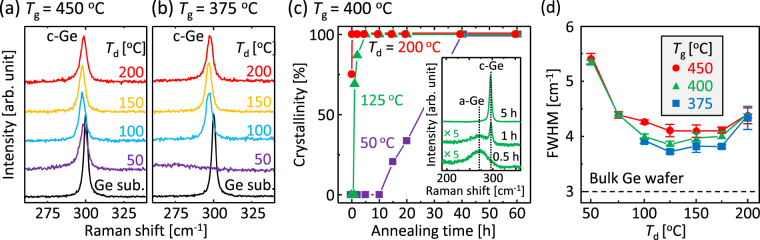
Raman spectroscopy study of the Ge layers. (**a**,**b**) Raman spectra of the sample annealed at (**a**) 450 °C for 5 h and (**b**) 375 °C for 140 h. The spectra for a bulk-Ge wafer are shown for comparison. (**c**) Crystallinity for samples with *T*_d_ = 50 °C, 125 °C, and 200 °C as a function of annealing time, determined by the annealing-time evolution of Raman spectra representatively shown in the insertion. (**d**) FWHMs of the Ge-Ge peaks for samples with *T*_g_ = 375 °C, 400 °C, and 450 °C as a function of *T*_d_. The data for a bulk-Ge wafer is shown by the dotted line.

**Figure 3:**
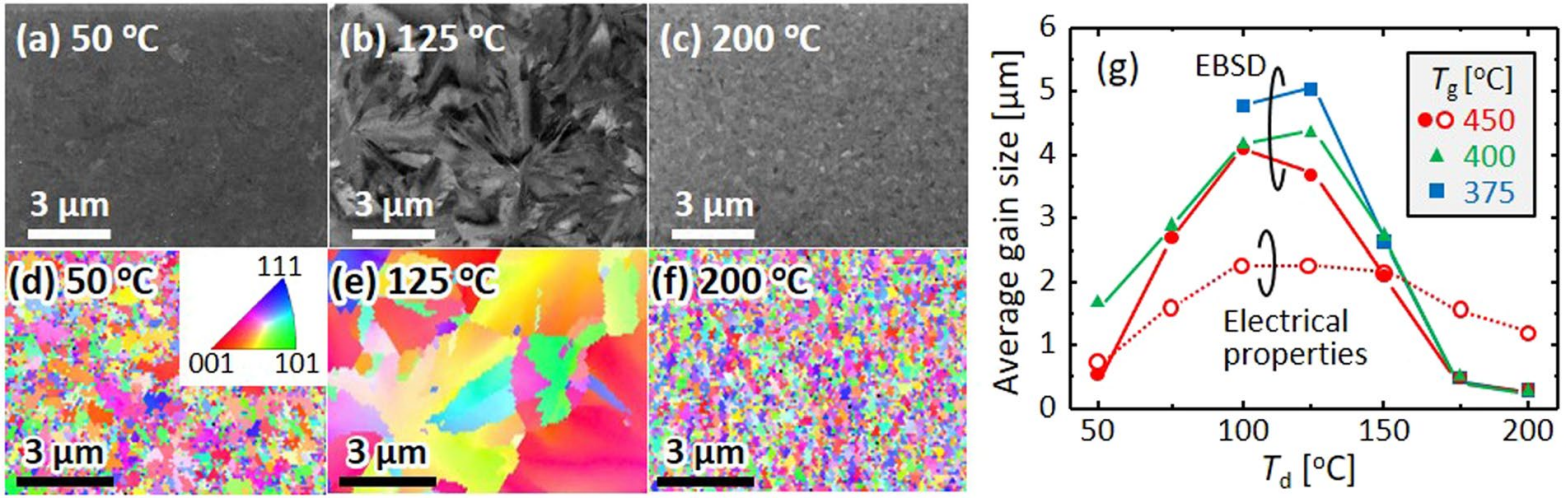
Grain size of the poly-Ge layers. (**a**–**c**) SEM and (**d–f**) EBSD images of the samples annealed at 450 °C with *T*_d_ = (**a,d**) 50 °C (**b,e**) 125 °C, and (**c,f**) 200 °C. The coloration in the EBSD images indicates the crystal orientation according to the legend inserted in (**d**). (**g**) Average grain size determined by the EBSD analyses for samples with *T*_g_ = 375 °C, 400 °C, and 450 °C as a function of *T*_d_. The data determined by the electrical properties of samples with *T*_g_ = 450 °C are also shown.

The samples were then annealed to induce SPC, where the growth temperature *T*_g_ ranged from 375 to 450 °C. The samples were evaluated by Raman spectroscopy. Figure 2a shows that all samples annealed at 450 °C for 5 h exhibited sharp peaks corresponding to crystal Ge. These peaks are almost symmetrical, suggesting that the Ge layers are completely crystallized. All peaks shifted to lower wavenumbers than that of a bulk Ge substrate, originating from the tensile strain. Generally, as-deposited amorphous semiconductor layers have stress caused by nanovoids or roughened surfaces^37,38^. This should affect the subsequent SPC; however, the initial stress is likely released during the crystallization of a-Ge. We therefore consider the tensile strain in the resulting Ge layers originates from the difference in the thermal expansion coefficients between Ge and the SiO_2_ substrate^17,23^. We note that samples with *T*_d_ = 100 and 150 °C exhibit larger tensile strains than other samples. This behaviour is likely caused by the large grain growth for samples with *T*_d_ = 100 and 150 °C, as revealed in Fig. 3. As representatively shown in Fig. 2b, annealing at 375 °C for 140 h crystallized the samples with *T*_d_ ≥ 100 °C, but not those with *T*_d_ ≤ 75 °C. We examined the *T*_d_ dependence of the growth rate from the annealing-time evolution of the Raman spectra, representatively shown in the insertion in Fig. 2c. The crystallinity of the Ge layer was defined as the ratio of the Raman peak intensity of c-Ge to that of a-Ge. Figure 2c shows that the annealing time for completing crystallization is dramatically reduced by increasing *T*_d_. This means that the higher *T*_d_ provides a higher growth rate, accounting for the results in Fig. 2b. The full width at half maximum (FWHM) of the Ge-Ge peaks were evaluated from the Raman spectra and are summarized in Fig. 2d. The FWHM depends on both *T*_d_ and *T*_g_: the FWHM is a minimum at approximately *T*_d_ = 125 °C and decreases with decreasing *T*_g_. The sample with *T*_d_ = 125 °C and *T*_g_ = 375 °C exhibited the minimum FWHM value (3.7 cm^−1^). This value is close to that of a bulk-Ge wafer (3.0 cm^−1^) and the smallest among poly-Ge formed at low temperatures (<600 °C)^17,18,23,30^. This indicates the high crystal quality of the Ge layer obtained in this study.

The grain size of the resulting poly-Ge layers was evaluated using scanning electron microscopy (SEM) and electron backscattering diffraction (EBSD) analyses. The SEM images in Fig. 3a–c show some contrast, especially for *T*_d_ = 125 °C. Atomic force microscopy (AFM) revealed that the surface of the sample with *T*_d_ = 125 °C was flat (root mean square value: 0.5 nm). This suggests that the contrast in SEM corresponds to crystal orientation visualized by the electron channelling effect for high-quality crystals. Figure 3d–f show that the crystal orientation is almost random for all samples. Both the SEM and EBSD images indicate that the sample with *T*_d_ = 125 °C exhibits grains that are one order of magnitude larger than those of the other samples. Figure 3g shows that the grain size, as determined by the EBSD analyses, depends on both *T*_d_ and *T*_g_. The grain size is a maximum at approximately *T*_d_ = 125 °C. The lower *T*_g_ provides a larger grain size, which agrees with the conventional SPC with^30^ and without^17^ catalytic metals. The dependence of the grain size on *T*_d_ and *T*_g_ accounts for the results of the Raman measurements shown in Fig. 2d. The sample with *T*_d_ = 125 °C and *T*_g_ = 375 °C exhibited a grain size of 5 µm, which is the largest among poly-Ge formed by SPC^17,18^.

**Figure 4:**
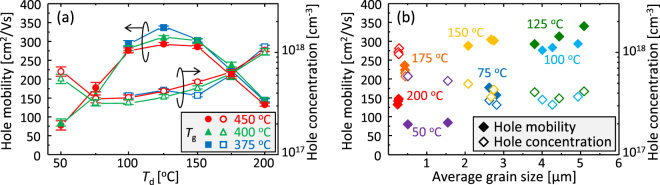
Electrical properties of the poly-Ge layers. Hole mobility and hole concentration for samples with *T*_g_ = 375 °C, 400 °C, and 450 °C as a function of (**a**) *T*_d_ and (**b**) average grain size.

The electrical properties of the resulting poly-Ge layers were evaluated by Hall effect measurements. All samples showed p-type conduction, similar to conventional non-doped poly-Ge^17,25,27,32^. This is because dangling bonds in Ge provide shallow acceptor levels and then generate holes at room temperature^39^. Figure 4a shows that the hole concentration depends on *T*_d_ and is a minimum at approximately *T*_d_ = 75–100 °C. Figure 4a also shows that the hole mobility depends on both *T*_d_ and *T*_g_, as with the grain size and FWHMs of the Raman peaks. The hole mobility is a maximum at *T*_d_ = 125 °C and increases with decreasing *T*_g_. The sample with *T*_d_ = 125 °C and *T*_g_ = 375 °C exhibited the highest hole mobility, 340 cm^2^/Vs. Figure 4b shows that the hole concentration decreases with increasing grain size, suggesting that dangling bonds in grain boundaries generate holes similar to those in crystal grains^6,10,17,39^. Figure 4b also shows that the hole mobility tends to increase with increasing grain size in the same *T*_d_, but overall, the hole mobility depends on *T*_d_ rather than grain size. For example, the sample with *T*_d_ = 175 °C exhibited higher hole mobility than the sample with *T*_d_ = 75 °C, whereas the grain size shows an opposing trend. This behaviour suggests that the hole mobility is not determined only by grain size, but also by another factor.

**Figure 5:**
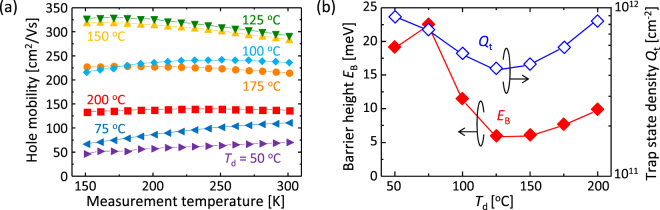
Characterization of grain boundaries in the poly-Ge layers. (**a**) Measurement temperature dependence of the hole mobility for samples with *T*_d_ = 50–200 °C and *T*_g_ = 450 °C. (**b**) Energy barrier height *E*_B_ and trap state density *Q*_t_ of grain boundaries for the samples with *T*_g_ = 450 °C as a function of *T*_d_.

According to the carrier conduction model in polycrystalline semiconductors proposed by Seto^40^, the carrier mobility limited by grain boundary scattering can be determined using the following equation:

1 $$\mu =\frac{Lq}{\sqrt{2\pi {m}^{\ast }kT}}{\rm{\exp }}\,(-\frac{{E}_{B}}{kT}),$$

where *µ* is the carrier mobility, *E*_B_ is the energy barrier height of the grain boundary, *T* is the absolute temperature, *L* is the grain size, m* is the effective mass, and *k* is the Boltzmann constant. Figure 5a shows that the *T* dependence of the hole mobility varies with *T*_d_, indicating the difference in *E*_B_^40^. The Arrhenius plot of *µT* ^1/2^ exhibited downward-sloping straight lines for all samples. This behaviour indicates that the carrier conduction in the poly-Ge layers is limited by grain boundary scattering. Figure 5b shows that *E*_B_, determined by the slopes of *µT* ^1/2^, depends on *T*_d_. For *T*_d_ = 125 °C, *E*_B_ exhibits the minimum value, 6.4 meV. The trap state density *Q*_t_ in the grain boundary can be determined using the following equation^40^:

2 $${Q}_{{\rm{t}}}=\frac{\sqrt{8\varepsilon N{E}_{B}}}{q},$$

where *N* is the carrier concentration, *ε* is the dielectric permittivity, and *q* is the elementary charge. Figure 5b shows that *Q*_t_ depends on *T*_d_. The *E*_B_ and *Q*_t_ for *T*_d_ = 50 °C nearly agree with those of conventional poly-Ge whose precursors were formed without heating the substrates^17,22,34^. For *T*_d_ = 125 °C, *Q*_t_ exhibits the minimum value, 4.4 × 10^11^ cm^−2^. From the equations (1) and (2), *μ* can be expressed by the following equation:

3 $$\mu =\frac{Lq}{\sqrt{2\pi {m}^{\ast }kT}}\,{\rm{\exp }}\,(-\frac{{q}^{2}{{Q}_{t}}^{2}}{8\varepsilon NkT}).$$

The equation (3) indicates that *Q*_t_ has greater influence on *μ* than *L*. Reflecting that, the *T*_d_ dependence of the hole mobility completely agrees with that of *Q*_t_ (Figs 4a and 5b). On the other hand, we determined *L* from the electrical properties of the sample with *T*_g_ = 450 °C by using the equation. The results are plotted in Fig. 3g, showing that the data from the electrical properties exhibits the same behaviour as that from the EBSD measurement. These results indicate that *μ* depends on not only *Q*_t_ but also *L*. We therefore conclude that the high hole mobility (340 cm^2^/Vs for *T*_d_ = 125 °C) observed in this study is due to both the large *L* and low *Q*_t_.

## Discussion

The crystal quality and electrical properties of the poly-Ge layer formed by SPC varied with the deposition temperature, *T*_d_, of the precursor Ge layer. According to Fig. 1 and the *T*_d_ dependent SPC properties, the precursor conditions can be determined by three regimes: the low-density regime (*T*_d_ < 100 °C), high-density regime (100 ≤ *T*_d_ ≤ 125 °C), and nucleation regime (*T*_d_ > 125 °C). In the low-density regime, the atomic distance in a-Ge is longer than that of crystal Ge. Therefore, the lateral growth rate of Ge nuclei during SPC should be low considering that SPC progresses by the migration of atoms during annealing. This speculation is consistent with the results on the growth rate shown in Fig. 2c. The low lateral growth rate result in poly-Ge with small grains because the grain size in SPC is determined by the balance between nucleation and lateral growth. When the atomic density of the a-Ge precursor is lower than that of crystalline Ge, the volume of the resulting SPC-Ge is smaller than that of the precursor. The volume change may cause the voids (dangling bonds) at the grain boundary. This leads to the high trap state density *Q*_t_ at grain boundaries and then lowers the hole mobility.

The nucleation regime should be *T*_d_ > 125 °C considering the behaviours of the grain size and electrical properties of the SPC-Ge. Although the sample with *T*_d_ = 150 °C exhibits no Raman peak (Fig. 1b), the Ge precursor layer may contain nuclei which are smaller or less than the detection limit of the Raman measurement. In the high-density regime, the atomic distance in the a-Ge precursor is close to that of crystalline Ge. The growth rate of Ge nuclei during SPC is therefore high, resulting in poly-Ge with large grains. The difference in the atomic density between the a-Ge precursor and resulting poly-Ge is small, leading to low *Q*_t_. The hole mobility is therefore high in the high-density regime. In the nucleation regime, the as-deposited Ge layer has dense crystalline Ge nuclei, which is common in vapor deposition^25,26^. Because SPC starts from the nuclei, the poly-Ge results in small grains. The nuclei perturb the atomic distance of the a-Ge region in the precursor, leading to high *Q*_t_. The hole mobility is therefore low in the nucleation regime. Thus, choosing a narrow window for *T*_d_ allows us to form the high-density amorphous precursor, which is essential for realizing high-quality poly-Ge. In previous studies, the effects of the deposition temperature of a-Si on the subsequent SPC were examined; however, the crystal quality and electrical properties were not improved dramatically^41^. This is probably because the substrate temperature was not set at the high-density regime but the nucleation regime considering that the grain size decreased with increasing *T*_d_^41^. We consider that the dramatic improvements of crystal quality and electrical properties occur for any materials as long as one can set the density of the amorphous precursors to the narrow high-density regime.

**Figure 6:**
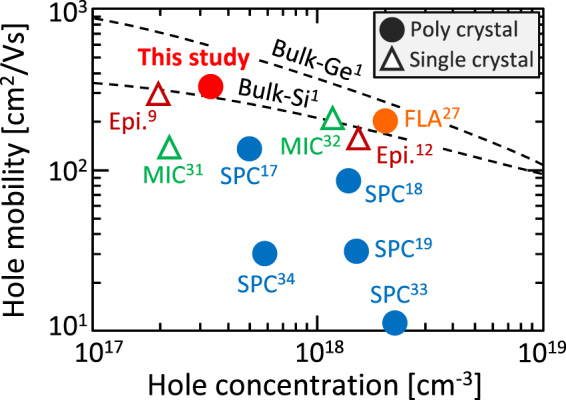
Comparison of the hole mobility and hole concentration of GOIs. The growth method and the reference number are shown near each symbol. The data for single-crystal bulk Si and Ge are shown by dotted lines.

As shown in Fig. 6, the hole concentration is the lowest among poly-Ge, indicating that the Ge layer contains relatively few defects. The hole mobility of 340 cm^2^/Vs obtained in this study is the highest value among the Ge layers formed on insulating substrates at temperatures below the melting point of Ge (937 °C). The hole mobility is even higher than that of single-crystal Ge layers epitaxially grown from single-crystal Si templates^9,12^. Moreover, the electrical properties of the present poly-Ge layer exceed those of bulk Si. These results mean that single-crystal wafers are no longer necessary for fabricating a high-hole mobility semiconductor thin film.

In conclusion, the condition of the precursor for SPC dramatically influences the crystal quality and electrical properties in the resulting poly-Ge layer. The a-Ge precursor prepared at 125 °C led to a large-grained (5 µm) poly-Ge layer whose grain boundary exhibited a low energy barrier height (6.4 meV) because of the low trap state density (4.4 × 10^11^ cm^−2^). This allowed for a hole mobility of 340 cm^2^/Vs, the highest value among semiconductor layers formed on insulators at temperatures below 900 °C. The growth temperature was as low as 375 °C, allowing for Ge devices on plastic substrates. These results open up the possibility for developing advanced CMOS devices as well as system-in-displays and three-dimensional integrated circuits. The findings from and knowledge gained in this study will be universal and applicable to various materials.

## Methods

### Sample preparation

The Ge precursors were deposited on SiO_2_ glass substrates using the Knudsen cell of a molecular beam deposition system (base pressure: 5 × 10^−7^ Pa). The deposition rate was 1.0 nm/min where the sample substrate was not heated. The deposition time was 100 min. The Ge source, manufactured by Furuuchi Chemical Corporation, had a purity of 99.999%. The substrate temperature during the deposition, *T*_d_, ranged from 50 to 200 °C. We note that *T*_d_ spontaneously rises from room temperature to 50 °C without heating the substrate because of the heat propagation from the Knudsen cell. The samples were then loaded into a conventional tube furnace in a N_2_ atmosphere and annealed at 375 °C for 140 h, 400 °C for 40 h, and 450 °C for 5 h to induce SPC.

### Material characterization

The XRR was performed using a Rigaku SmartLab. The Raman spectroscopy was performed using a Nanophoton RAMANplus, where the laser wavelength was 532 nm and the spot size was 20 µm. The SEM and EBSD analyses were performed using a JEOL JSM-7001F with a TSL OIM analysis attachment. The AFM was performed using a Shimadzu SPM-9600. The Hall effect measurement with the Van der Pauw method was performed using a Bio-Rad HL5500PC. The hole mobility and hole concentration were averaged over five measurements for each sample.
